# Impact of H19 Polymorphisms on Prostate Cancer Clinicopathologic Characteristics

**DOI:** 10.3390/diagnostics10090656

**Published:** 2020-08-31

**Authors:** Ju-Chuan Hu, Chia-Yen Lin, Shian-Shiang Wang, Kun-Yuan Chiu, Jian-Ri Li, Chuan-Shu Chen, Sheng-Chun Hung, Cheng-Kuang Yang, Yen-Chuan Ou, Chen-Li Cheng, Shun-Fa Yang

**Affiliations:** 1 Institute of Medicine, Chung Shan Medical University, Taichung 402, Taiwan; shelleycain525@gmail.com (J.-C.H.); lcyhank.tw@gmail.com (C.-Y.L.); sswdoc@yahoo.com.tw (S.-S.W.); fisherfishli@yahoo.com.tw (J.-R.L.); r2060d@yahoo.com.tw (C.-S.C.); weshong1118@gmail.com (S.-C.H.); ycou228@gmail.com (Y.-C.O.); cheng20011@gmail.com (C.-L.C.); 2 Division of Urology, Department of Surgery, Taichung Veterans General Hospital, Taichung 407, Taiwan; chiu37782002@yahoo.com (K.-Y.C.); yangck@vghtc.gov.tw (C.-K.Y.); 3 Division of Urology, Department of Surgery, Chiayi Branch, Taichung Veterans General Hospital, Chiayi 600, Taiwan; 4 Department of Applied Chemistry, National Chi Nan University, Nantou 545, Taiwan; 5 Department of Medicine and Nursing, Hung Kuang University, Taichung 433, Taiwan; 6 Department of Urology, Tung’s Taichung MetroHarbor Hospital, Taichung 433, Taiwan; 7 Department of Medical Research, Chung Shan Medical University Hospital, Taichung 402, Taiwan

**Keywords:** H19, prostate cancer, polymorphism, outcome

## Abstract

Active surveillance is the preferred strategy for very low risk, low risk, and some favorable intermediate risk of prostate cancer. However, the current risk stratifications with initial prostate-specific antigen (iPSA) levels and Gleason scores at biopsy can underestimate the true oncologic threat. More precise predictors are required to avoid the overtreatment of prostate cancer. H19 single-nucleotide polymorphisms (SNPs) have been found to play crucial roles in numerous malignancies, but not yet in prostate cancer. This study assessed the clinicopathologic effects of H19 SNPs on prostate cancer to identify potential active surveillance candidates. A total of 579 patients with prostate cancer who underwent robot-assisted radical prostatectomy between 2012 and 2017 were recruited. The patients were grouped by iPSA levels, and five H19 SNPs were evaluated. Our results show that patients with an iPSA level of ≤7 ng/mL had increased an likelihood of having Gleason score and group grade upgrades after radical prostatectomy compared with patients with an iPSA level of >7 ng/mL. Moreover, patients with loci polymorphisms in either rs3024270 or rs3741219 had a significantly higher risk of perineural invasion (rs3024270: Odds ratio (OR) 2.76, 95% confidence interval (CI) 1.30–5.87, *p* = 0.01; rs3741219: OR 2.30, 95% CI 1.17–4.54, *p* = 0.018). In conclusion, our results suggested that H19 SNPs play a role in the perineural invasion of prostate cancer.

## 1. Introduction

In 2018, prostate cancer was the second most common cancer in men and the fifth major cause of death worldwide [1,2]. As the diagnostic rate of prostate cancer increases, its associated mortality decreases [2,3]. The wide application of prostate-specific antigen (PSA) screening in many countries is the main cause of the increased detection and reduced cancer-specific death [4,5,6]. However, overdiagnosis, overtreatment, and subsequent adverse effects of curative treatments for prostate cancer impair patients’ quality of life and increase public health expenditure [6]. For patients who are classified as having a very low risk and low risk of prostate cancer, active surveillance (AS) is the preferred therapeutic strategy to avoid complications related to radical prostatectomy or radiotherapy, such as urinary incontinence and sexual dysfunction [6,7,8]. The risk group grading system was developed in the 2014 International Society of Urological Pathology Consensus Conference, and has been validated by several large-scale studies [9,10]. However, the risk of oncologic progression may be underestimated based on only the results of random prostate biopsy and the initial PSA (iPSA) level, resulting in an undesirable delay for initiating definite treatment. Caster et al. compared the Gleason score at biopsy among 25,858 patients with prostate cancer with their pathological Gleason score after radical prostatectomy. An upgrade in Gleason score was seen in 43% of patients with a biopsy Gleason score of 6 and a PSA of <10 ng/mL [11]. Hence, more accurate tools for evaluating oncologic risks are required for developing individually tailored therapies for prostate cancer, especially when AS is considered.

Oncogenesis is associated with epigenetic and genetic alterations [12]. An estimated 89% of metastatic castration-resistant prostate cancers carry an essentially actionable mutation, and some of these are germline mutations [13]. The genetic variations could act as markers to enhance cancer risk assessment. Several genetic mutations, including Breast Cancer genes 2 (BRCA2) and BRCA1, have been proven to be strongly associated with prostate cancer [14,15,16]. Long noncoding ribonucleic acids (lncRNAs) play multiple roles in the regulation of various biological processes and cancer pathogenesis [17,18,19]. A well-known lncRNA used as a diagnostic tool for prostate cancer is prostate cancer gene 3 (PCA3), which is located in chromosome 9q21-22 [20]. Many lncRNAs are essential in the pathophysiology of prostate cancer, irrespective of the interplay with the androgen receptor [21,22,23].

LncRNA H19, which is located in 11p15.5, is upregulated in various human solid tumors, including bladder cancer, non–small cell lung cancer, breast cancer, and gastric cancer [24,25,26]. Although evidence suggests that H19 functions as an oncogene, its expression and regulatory mechanism in prostate cancer remain controversial [27,28,29]. Single-nucleotide polymorphisms (SNPs) in the H19 gene are associated with cancer susceptibilities and clinicopathologic features [30,31,32,33,34]. However, limited data on the associations between H19 SNPs and prostate cancer are available. The present study assessed how H19 polymorphisms interplay with the clinicopathologic characteristics of prostate cancer, especially in patients with relatively low iPSA levels (low risk of prostate cancer) to identify the best candidates for curative treatments.

## 2. Materials and Methods

### 2.1. Study Population

A total of 579 patients with adenocarcinoma of the prostate were recruited in this hospital-based study. All the patients received robot-assisted radical prostatectomy with bilateral pelvic lymph node dissection between 2012 and 2017 at Taichung Veteran General Hospital. The study was approved by the Institutional Review Board of Taichung Veteran General Hospital (IRB No. CE19062A; 04/March/2019), and written informed consent was obtained from all the participants.

Medical information was gathered through a chart review, including the iPSA levels at diagnosis, the clinical and pathologic tumor-node-metastasis (TNM) stage, the D’Amico classification [35], the pathologic Gleason grade group at biopsy and at radical prostatectomy, and other pathologic presentations. The patients were divided into three groups by their iPSA level at diagnosis (≤7, 7–10, and >10 ng/mL) to evaluate patients with low risks of prostate cancer [35,36].

### 2.2. Specimen Collection and DNA Extraction

Whole-blood samples were collected through venipuncture from 579 patients before the surgery and were stored in the ethylenediaminetetraacetic acid-coated tubes. DNA extraction was performed using the QIAamp DNA Blood Mini Kits (Qiagen, Valencia, CA, USA), according to the manufacturer’s protocol. Five H19 genetic polymorphisms (rs2177727, rs2107425, rs2839698, rs3024270, and rs3741219) were selected based on the data of the International HapMap Project dbSNP database and previous works [31,37]. The allelic variants were assessed using an ABI StepOne Real-Time PCR System (Applied Biosystems, Foster City, CA, USA) and analyzed using a TaqMan Assay with the SDS 3.0 software (Applied Biosystems). The detailed methods of DNA extraction were similar to those in our previous study [32,33,34].

### 2.3. Statistical Analysis

The adjusted odds ratio (AOR) with a 95% confidence interval (CI) was assessed using multiple logistic regression after adjustment for other covariates to determine the associations between the H19 genotypic frequencies and the iPSA level. The odds ratios (ORs) of genotypic frequencies in different clinicopathologic features were analyzed using the logistic regression model. *p* < 0.05 was set as statistically significant. The data were analyzed with the SAS statistical software (version 9.1 for Windows; SAS Institute, Cary, NC, USA).

## 3. Results

### 3.1. Characteristics of Study Participants

The distributions of the demographic characteristics of patients in the three PSA groups (153 patients with an iPSA level of ≤ 7 ng/mL, 117 with an iPSA level of 7–10 ng/mL, and 309 with an iPSA level of > 10 ng/mL) are presented in Table 1. The iPSA > 10 ng/mL group had more patients with adverse pathologic features, including the Gleason grade group 4+5, pathologic T stage 3+4, extraprostatic extension, seminal vesicle invasion, and perineural invasion (PNI). The clinical TNM stages were similar between the iPSA ≤ 7 ng/mL and iPSA 7–10 ng/mL groups, and the incidence of advanced pathological T stage and positive pathologic features such as extraprostatic extension, seminal vesicle invasion, and lymphovascular invasion were obviously lower in the iPSA ≤ 7 ng/mL group. However, this group also had higher rates of Gleason score and grade group upgrades after radical prostatectomy than the iPSA 7–10 ng/mL group (Gleason score upgrade, 43.1% vs. 36.8%, *p* = 0.29; Gleason grade group upgrade, 45.1% vs. 38.5%, *p* = 0.27, respectively).

### 3.2. Association of H19 Polymorphisms with Risk and Clinical Features of Prostate Cancer

The distribution frequencies of the five H19 genotypes (rs2177727, rs2107425, rs2839698, rs3024270, and rs3741219) are presented in Table 2. The AORs with a 95% CI were analyzed using multiple logistic regression models after the adjustment of other covariates, including age, pathological Gleason grade group, clinical TNM stage, pathologic T and *n* stage, seminal vesicle invasion, PNI, lymphovascular invasion, and D’Amico classification. The results revealed that rs3024270 and rs3741219 were nonsignificantly higher in the iPSA ≤7 ng/mL group. The other three loci (rs2177727, rs2107425, and rs2839698) did not show significant differences in their genotypic frequencies. Accordingly, we analyzed the associations between these two genotypic frequencies and the clinicopathologic attributes in the iPSA ≤7 ng/mL group (Table 3 and Table 4). The patients with loci polymorphisms in either rs3024270 or rs3741219 had a significantly higher risk of PNI (rs3024270: OR 2.76, 95% CI 1.30–5.87, *p* = 0.01; rs3741219: OR 2.30, 95% CI 1.17–4.54, *p* = 0.018). Additionally, the patients with the rs3024270 polymorphism had significantly higher occurrences of positive extraprostatic extension (OR 4.89, 95% CI 1.62–14.74). However, the presence of H19 SNPs was not associated with the Gleason score or grade group upgrade, biochemical recurrence, and overall survival in these patients. In addition, there were no significant associations between H19 rs3024270 or rs3741219 polymorphisms and the clinicopathologic features in the 7–10 ng/mL iPSA group and the >10 ng/mL iPSA group (data not shown).

### 3.3. Association between H19 mRNA Expression and Clinical Characteristics of Prostate Cancer from TCGA Database

**Figure 1:**
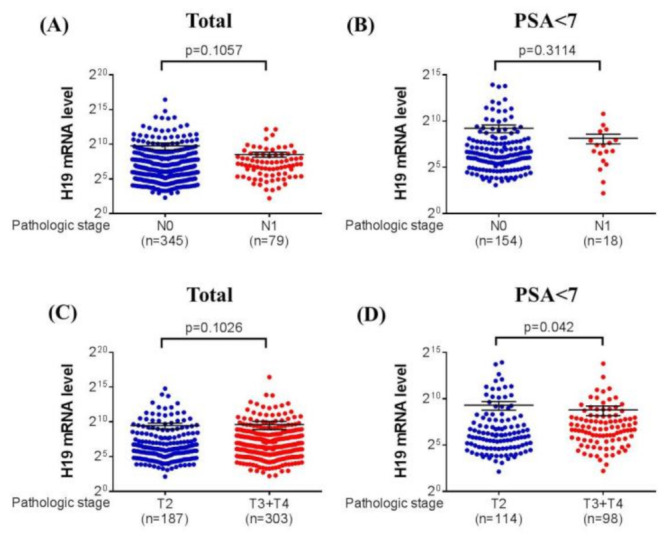
H19 mRNA level of patients with prostate cancer from the Cancer Genome Atlas (TCGA) database. (**A**) H19 mRNA levels were compared according to the lymph node status in the whole population. (**B**) H19 mRNA levels were compared according to the lymph node status in the initial PSA (iPSA) < 7 group. (**C**) H19 mRNA levels were compared according to the pathological T stage in the whole population. (**D**) H19 mRNA levels were compared according to the pathological T stage in the iPSA < 7 group.

We examined the associations between the H19 mRNA level and the pathologic tumor and nodal stage according to the baseline PSA level by accessing The Cancer Genome Atlas (TCGA) database (Figure 1). The results revealed a consistent trend that cases with node-positive disease had lower H19 mRNA expression, regardless of their baseline PSA level. In the iPSA < 7 ng/mL group, the H19 mRNA level was significantly lower in patients in advanced tumor stages than in those in the T2 stage (*p* = 0.042).

## 4. Discussion

To the best of our knowledge, this is the first study to evaluate H19 polymorphism and its associations with the clinicopathologic presentations of prostate cancer among Taiwanese men, especially focused on patients with relatively low iPSA levels. Patients with an iPSA level of ≤ 7 ng/mL with the rs3024270 or rs3741219 polymorphism had a more than twofold increase in PNI incidence, and those with the rs3024270 variation experienced a 4.89-fold increased risk of extraprostatic extension. Although the presence of H19 polymorphisms was positively associated with adverse pathologic features, the H19 mRNA level was significantly lower in patients in the advanced T stage than those in the T2 stage when their iPSA was ≤ 7 ng/mL in the TCGA database. These findings suggest that the lncRNA H19 might have more complex interactions with prostate cancer than merely acting as an oncogene.

Overdiagnosis and overtreatment remain an unresolved issue in prostate cancer. Various biomarker assays have been validated for their diagnostic efficacies in clinically significant prostate cancer. Currently, FDA-approved assessments, including Progensa-PCA3 and prostate health index (PHI) tests, provide informative recommendations for repeated prostate biopsy among men with a previous negative biopsy [20,38,39,40]. The PHI test is also helpful in both reducing unnecessary initial biopsies in biopsy-naïve patients and predicting the pathological aggressiveness at radical prostatectomy [40]. Despite the above well-established instruments, we hope to identify additional biomarkers to improve the decision-making strategies. Therefore, risk SNPs extracted from blood samples might play a potential role in implementing curative treatment.

We grouped the participants with an iPSA cutoff level of 7 ng/mL in addition to a traditional D’Amico low-risk classification for several reasons [35]. The major concern is reassuring the patients with relatively low iPSA levels that AS is not only the optimal but also a safe strategy. Traditional risk categorizations such as D’Amico classification based on the iPSA level and Gleason score at biopsy might underestimate the oncologic severity and risk of disease upgrade [11]. One study evaluated patients with negative results at their first prostate biopsy to identify potential predictors for prostate cancer [36]. In patients with prostate cancer diagnosed at the second biopsy, the mean PSA level at the first biopsy was 6.44 ng/mL, whereas patients with two continuously negative biopsies had a mean PSA level of 7.63 ng/mL at the second prostate biopsy. Therefore, a PSA cutoff level of 7 ng/mL seemed reasonable for exploring the predispositions of risk SNPs for prostate cancer, reducing treatment-related complications, and reducing public health burdens.

Nowadays, D’Amico classification based on PSA level, biopsy Gleason score, and the clinical stage seems insufficient to identify potential high-risk patients from actual low-risk groups. Several potential predictors were applied in the evaluation of oncologic prognosis. The inflammatory responses interfere with the tumor microenvironment. Thus, some laboratory markers such as the neutrophil-to-lymphocyte ratio and platelet-to-lymphocyte ratio were remarkable predictors for Gleason score upgrading at radical prostatectomy [41]. The other promising factors associated with the oncologic outcome were androgen-associated markers. Ferro at al. recently found that the circulating testosterone level had a potential impact on unfavorable disease, including an International Society of Urological Pathology (ISUP) grade group ≥ 3 and/or pT ≥ 3a [42]. The last indicative factors for the progression of prostate cancer involved plenty of genetic-associated biomarkers.

The development of prostate cancer is associated with acquired and inherited factors. lncRNA and its genotypic variations have diverse effects on the development of prostate cancer [43,44]. Our previous study proved that lncRNA growth arrest-specific 5 (GAS5) polymorphism could be a predictor for lymph node metastasis [45]. H19, as the first discovered lncRNA, was found in the 1980s, and the association between its polymorphism and bladder cancer was identified in 2008 [31]. At present, many SNPs in H19 have been reported to be strongly associated with cancer susceptibilities, particularly polymorphisms at rs2839698, rs2107425, and rs2177727 [30,31,32,33,34]. However, the data are limited for connections between H19 SNPs and prostate cancer, especially regarding variations at rs3024270 and rs3741219. The present study demonstrated that rs3024270 and rs3741219 were significantly related to increased risks of PNI after radical prostatectomy in patients with iPSA of ≤ 7 ng/mL.

PNI is a sign of tumor metastasis and indicates poor prognosis in numerous cancers [46]. For prostate cancer, PNI is an independent prognostic indicator of adverse survival outcomes, particularly for patients with National Comprehensive Cancer Network (NCCN) low risk of disease [47,48]. One landmark study revealed that five high-risk SNPs and a positive family history were estimated to comprise 46% of prostate cancer cases [49]. Indeed, a combination of SNPs and PSA levels may enhance the risk estimation of prostate cancer [50,51]. Similarly, our findings indicate that H19 SNPs could be a helpful predictor in identifying cases with adverse pathologic outcomes when there is iPSA of ≤7 ng/mL.

Studies have indicated that H19 might act as an oncogene in prostate cancer by the upregulation of SRY box 2 (SOX2) and POU domain class 5 transcription factor 1 (POU5F1) [23,29]. However, its expression pattern remains uncertain in prostate cancer [52]. We found that the H19 mRNA expression was significantly lower in patients with an advanced tumor stage when they had iPSA of ≤ 7 ng/mL in the TCGA database. Taken together, these findings suggested that H19 might play both promotive and inhibitory roles in prostate carcinogenesis.

The present study has a few limitations. First, only patients who received radical prostatectomy were enrolled, but these patients were relatively younger (mean age, 67.1 years). They were also more likely to have less advanced diseases and a better performance status. Second, the follow-up periods might be relatively insufficient to observe biochemical recurrence and cancer-specific mortality among patients with a iPSA level of ≤ 7 ng/mL. Third, only Taiwanese populations were involved; hence, ethnic differences cannot be ruled out. These limitations might underestimate the risks of H19 SNPs in prostate cancer prognosis. Furthermore, the sample size in this study was relatively small. Finally, a critical limitation is the retrospective design of the present study, although data were derived from our prospectively collected database.

## 5. Conclusions

In conclusion, patients with a relatively low iPSA level (≤7 ng/mL) had increased risks of Gleason score and grade group upgrade after radical prostatectomy compared with patients with an iPSA level of >7 ng/mL. Moreover, these patients experienced significantly higher risks of positive PNI and/or extraprostatic extension if presenting with H19 polymorphisms at rs3024270 and rs3741219. Although no significant associations between the H19 polymorphisms, biochemical recurrence, and overall survival were identified, the results might be underestimated due to the study limitations. Further large-scale studies are warranted to examine the roles of regulatory lncRNA H19 in prostate cancer.

## Figures and Tables

**Table 1:** The distributions of demographical characteristics in 579 patients with prostate cancer.

Variable	PSA at Diagnosis (ng/mL)		
	<7 (*n* = 153)	7–10 (*n* = 117)	>10 (*n* = 309)
**Age at diagnosis (years)**			
<65	71 (46.4%)	61 (52.1%)	113 (36.6%)
>65	82 (53.6%)	56 (47.9%)	196 (63.4%)
**Pathologic Gleason grade group**			
1 + 2 + 3	142 (92.8%)	108 (92.3%)	234 (75.7%)
4 + 5	11 (7.2%)	9 (7.7%)	75 (24.3%)
**Clinical T stage**			
1 + 2	144 (94.1%)	109 (93.2%)	248 (80.3%)
3 + 4	9 (5.9%)	8 (6.8%)	61 (19.7%)
**Clinical *n* stage**			
N0	152 (99.3%)	115 (98.3%)	299 (96.8%)
N1	1 (0.7%)	2 (1.7%)	10 (3.2%)
**Clinical M stage**			
M0	153 (100.0%)	117 (100.0%)	299 (96.8%)
M1	0 (0.0%)	0 (0.0%)	10 (3.2%)
**Pathologic T stage**			
2	114 (74.5%)	72 (61.5%)	120 (38.8%)
3 + 4	39 (25.5%)	45 (38.5%)	189 (61.2%)
**Pathologic *n* stage**			
N0	146 (95.4%)	113 (96.6%)	384 (87.7%)
N1	7 (4.6%)	4 (3.4%)	38 (12.3%)
**Extraprostatic extension**			
No	107 (69.9%)	71 (60.7%)	148 (47.9%)
Yes	46 (30.1%)	46 (39.3%)	161 (52.1%)
**Seminal vesicle invasion**			
No	145 (94.8%)	99 (84.6%)	208 (67.3%)
Yes	8 (5.2%)	18 (15.4%)	101 (32.7%)
**Perineural invasion**			
No	53 (34.6%)	40 (34.2%)	62 (20.1%)
Yes	100 (65.4%)	77 (65.8%)	247 (79.9%)
**Lymphovascular invasion**			
No	145 (94.8%)	101 (86.3%)	236 (76.4%)
Yes	8 (5.2%)	16 (13.7%)	73 (23.6%)
**D’Amico classification**			
Low risk	41 (26.8%)	19 (16.2%)	0 (0.0%)
Intermediate risk	64 (41.8%)	66 (56.4%)	90 (29.1%)
High risk	48 (31.4%)	32 (27.4%)	219 (70.9%)

**Table 2:** Distribution frequency of the *H19* genotypes in 579 patients with prostate cancer.

Variable	PSA at Diagnosis (ng/mL)				
	<7 (*n* = 153)	7–10 (*n* = 117)	>10 (*n* = 309)	AOR (95% CI) ^a^	AOR (95% CI) ^b^
**rs2177727**					
CC	69 (45.1%)	47 (40.2%)	133 (43.0%)	1.00	1.00
CT	71 (46.4%)	50 (42.7%)	140 (45.3%)	0.832 (0.475–1.460)	0.894 (0.553–1.445)
TT	13 (8.5%)	20 (17.1%)	36 (11.7%)	2.197 (0.971–4.971)	1.297 (0.583–2.887)
CT+TT	84 (54.9%)	70 (59.8%)	176 (57.0%)	1.054 (0.627–1.771)	0.960 (0.609–1.514)
**rs2107425**					
CC	57 (37.3%)	41 (35.0%)	111 (35.9%)	1.00	1.00
CT	75 (49.0%)	52 (44.4%)	143 (46.3%)	0.839 (0.476–1.480)	0.879 (0.535–1.446)
TT	21 (13.7%)	24 (20.5%)	55 (17.8%)	1.537 (0.731–3.231)	1.121 (0.564–2.230)
CT+TT	96 (62.7%)	76 (65.0%)	198 (64.1%)	0.991 (0.584–1.681)	0.934 (0.584–1.494)
**rs2839698**					
CC	74 (48.4%)	56 (47.9%)	149 (48.2%)	1.00	1.00
CT	65 (42.5%)	53 (45.3%)	135 (43.7%)	1.063 (0.628–1.799)	1.108 (0.691–1.777)
TT	14 (9.2%)	8 (6.8%)	25 (8.1%)	0.915 (0.343–2.441)	1.064 (0.467–2.424)
CT+TT	79 (51.6%)	61 (52.1%)	160 (51.8%)	1.040 (0.627–1.724)	1.100 (0.701–1.728)
**rs3024270**					
CC	38 (24.8%)	37 (31.6%)	92 (29.8%)	1.00	1.00
CG	84 (54.9%)	55 (47.0%)	144 (46.6%)	0.595 (0.324–1.092)	0.791 (0.463–1.351)
GG	31 (20.3%)	25 (21.4%)	73 (23.6%)	0.934 (0.449–1.941)	1.040 (0.543–1.991)
CG+GG	115 (75.2%)	80 (68.4%)	217 (70.2%)	0.683 (0.387–1.206)	0.860 (0.518–1.427)
**rs3741219**					
AA	69 (45.1%)	55 (47.0%)	146 (47.2%)	1.00	1.00
AG	67 (43.8%)	53 (45.3%)	135 (43.7%)	0.977 (0.575–1.661)	1.001 (0.622–1.611)
GG	17 (11.1%)	9 (7.7%)	28 (9.1%)	0.819 (0.325–2.068)	0.896 (0.408–1.967)
AG+GG	84 (54.9%)	62 (53.0%)	163 (52.8%)	0.949 (0.571–1.579)	0.981 (0.623–1.544)

The adjusted odds ratios (AORs) with their 95% confidence intervals (CIs) were estimated by multiple logistic regression models after controlling for age at diagnosis, pathologic Gleason grade group, clinical T stage, clinical *n* stage, clinical M stage, pathologic T stage, pathologic *n* stage, extraprostatic extension, seminal vesicle invasion, perineural invasion, lymphovascular invasion, and D’Amico classification. ^a^ AORs with their 95% CIs were calculated between patients with a PSA level of <7 ng/mL and PSA level of 7–10 ng/mL. ^b^ AORs with their 95% CIs were calculated between patients with a PSA level of <7 ng/mL and PSA level of >10 ng/mL.

**Table 3:** Odds ratio (OR) and 95% confidence interval (CI) of the clinical status and *H19* rs3024270 genotypic frequencies in 153 patients with prostate cancer with a PSA concentration under 7 ng/mL.

Variable	Genotypic Frequencies			
rs3024270	CC (*n* = 38)	CG+GG (*n* = 115)	OR (95% CI)	*p* Value
**Pathologic Gleason grade group**				
1 + 2 + 3	36 (94.7%)	106 (92.2%)	1.00	*p* = 0.732
4 + 5	2 (5.3%)	9 (7.8%)	1.528 (0.315–7.406)	
**Clinical T stage**				
1 + 2	34 (89.5%)	110 (95.7%)	1.00	*p* = 0.227
3 + 4	4 (10.5%)	5 (4.3%)	0.386 (0.098–1.520)	
**Pathologic T stage**				
2	33 (86.8%)	81 (70.4%)	1.00	*p* = 0.053
3 + 4	5 (13.2%)	34 (29.6%)	2.770 (0.997–7.701)	
**Pathologic *n* stage**				
N0	37 (97.4%)	109 (94.8%)	1.00	*p* = 0.682
N1	1 (2.6%)	6 (5.2%)	2.037 (0.237–17.478)	
**Extraprostatic extension**				
No	34 (89.5%)	73 (63.5%)	1.00	*p* = 0.002 *
Yes	4 (10.5%)	42 (36.5%)	4.890 (1.622–14.741)	
**Seminal vesicle invasion**				
No	37 (97.4%)	108 (93.9%)	1.00	*p* = 0.680
Yes	1 (2.6%)	7 (6.1%)	2.398 (0.285–20.146)	
**Perineural invasion**				
No	20 (52.6%)	33 (28.7%)	1.00	*p* = 0.010 *
Yes	18 (47.4%)	82 (71.3%)	2.761 (1.299–5.869)	
**Lymphovascular invasion**				
No	37 (97.4%)	108 (93.9%)	1.00	*p* = 0.680
Yes	1 (2.6%)	7 (6.1%)	2.398 (0.285–20.146)	
**D’Amico classification**				
Low/Intermediate risk	24 (63.2%)	81 (70.4%)	1.00	*p* = 0.425
High risk	14 (36.8%)	34 (29.6%)	0.720 (0.333–1.556)	
**Total score upgrade**				
No	21 (55.3%)	66 (57.4%)	1.00	*p* = 0.852
Yes	17 (44.7%)	49 (42.6%)	0.917 (0.438–1.919)	
**Grade group upgrade**				
No	20 (52.6%)	64 (55.7%)	1.00	*p* = 0.851
Yes	18 (47.4%)	51 (44.3%)	0.885 (0.424–1.847)	

The ORs analyzed by their 95% CIs were estimated by logistic regression models. * *p* value < 0.05 is statistically significant.

**Table 4:** Odds ratio (OR) and 95% confidence interval (CI) of clinical status and *H19* rs3741219 genotypic frequencies in 153 patients with prostate cancer with a PSA concentration under 7 ng/mL.

Variable	Genotypic Frequencies			
rs3741219	AA (*n* = 69)	AG+GG (*n* = 84)	OR (95% CI)	*p* Value
**Pathologic Gleason grade group**				
1 + 2 + 3	64 (92.8%)	78 (92.9%)	1.00	*p* = 0.610
4 + 5	5 (7.2%)	6 (7.1%)	0.985 (0.287–3.375)	
**Clinical T stage**				
1 + 2	64 (92.8%)	80 (95.2%)	1.00	*p* = 0.732
3 + 4	5 (7.2%)	4 (4.8%)	0.640 (0.165–2.482)	
**Pathologic T stage**				
2	55 (79.7%)	59 (70.2%)	1.00	*p* = 0.197
3 + 4	14 (20.3%)	25 (29.8%)	1.665 (0.287–3.375)	
**Pathologic *n* stage**				
N0	65 (94.2%)	81 (96.4%)	1.00	*p* = 0.702
N1	4 (5.8%)	3 (3.6%)	0.602 (0.130–2.785)	
**Extraprostatic extension**				
No	48 (69.6%)	59 (70.2%)	1.00	*p* = 0.534
Yes	21 (30.4%)	25 (29.8%)	0.969 (0.484–1.939)	
**Seminal vesicle invasion**				
No	64 (92.8%)	81 (96.4%)	1.00	*p* = 0.469
Yes	5 (7.2%)	3 (3.6%)	0.474 (0.109–2.059)	
**Perineural invasion**				
No	31 (44.9%)	22 (26.2%)	1.00	*p* = 0.018 *
Yes	38 (55.1%)	62 (73.8%)	2.299 (1.165–4.535)	
**Lymphovascular invasion**				
No	64 (92.8%)	81 (96.4%)	1.00	*p* = 0.469
Yes	5 (7.2%)	3 (3.6%)	0.474 (0.109–2.059)	
**D’Amico classification**				
Low/Intermediate risk	46 (66.7%)	59 (70.2%)	1.00	*p* = 0.727
High risk	23 (33.3%)	25 (29.8%)	0.847 (0.427–1.681)	
**Total score upgrade**				
No	42 (60.9%)	45 (53.6%)	1.00	*p* = 0.414
Yes	27 (39.1%)	39 (46.4%)	1.348 (0.706–2.573)	
**Grade group upgrade**				
No	40 (58.0%)	44 (52.4%)	1.00	*p* = 0.517
Yes	29 (42.0%)	40 (47.6%)	1.254 (0.600–2.382)	

The ORs analyzed by their 95% CIs were estimated by logistic regression models. * *p* value < 0.05 is statistically significant.
